# Altered Temporal Dynamics of the Amplitude of Low-Frequency Fluctuations in Comitant Exotropia Patients

**DOI:** 10.3389/fnhum.2022.944100

**Published:** 2022-07-13

**Authors:** Ri-Bo Chen, Shu-Yuan Ye, Chong-Gang Pei, Yu-Lin Zhong

**Affiliations:** ^1^Department of Radiology, Jiangxi Provincial People's Hospital, The First Affiliated Hospital of Nanchang Medical College, Nanchang, China; ^2^Department of Ophthalmology, The First Affiliated of Nanchang University, Nanchang, China; ^3^Department of Ophthalmology, Jiangxi Provincial People's Hospital, The First Affiliated Hospital of Nanchang Medical College, Nanchang, China

**Keywords:** comitant exotropia, functional magnetic resonance imaging, dynamic amplitude of low-frequency fluctuation, stereoscopic vision, support vector machine

## Abstract

**Purpose:**

Growing evidence reported that patients with comitant exotropia (CE) were accompanied by static cerebral neural activity changes. However, whether the dynamic time-varying of neural activity changes in patients with CE remains unknown.

**Methods:**

A total of 36 patients with CE (25 men and 11 women) and 36 well-matched healthy controls are enrolled in the study. The dynamic amplitude of low-frequency fluctuation (dALFF) combined with the sliding window method was used to assess the dynamic neural activity changes in patients with CE.

**Results:**

Compared with HCs, patients with CE had decreased dALFF values in the right superior parietal lobule (SPL) and right precuneus gyrus (PreCUN). Moreover, we found that the dALFF maps showed an accuracy of 48.61% and an area under the curve of.54 for distinguishing the patients with CE from HCs.

**Conclusion:**

Our study demonstrated that patients with CE showed altered dynamic neural activity changes in the right SPL and right PreCUN, which might indicate the neuropathological mechanism of stereoscopic dysfunction in patients with CE.

## Introduction

Comitant exotropia (CE) is a common ophthalmic disease. Patients with CE were associated with impaired stereoscopic vision. At present, the surgical treatment of strabismus correction is an important treatment for patients with CE. However, there are some strabismus patients who cannot reconstruct the stereoscopic vision completely, which had a bad influence on their daily life of these patients. Recent studies have shown that strabismus patients are more likely to be accompanied by emotional and psychological abnormalities (Lin et al., 2014; McBain et al., 2014; Lee et al., 2022). However, the exact mechanism of brain pathology in strabismus patients remains unclear.

Recently, the fMRI technology can be successfully used to detect static cerebral neural activity changes. Moreover, fMRI has been widely used to detect neural activity changes in strabismus patients. Shi et al. (2019) reported that constant exotropia patients had lower regional homogeneity (ReHo) values in the right secondary visual cortex (V2). Xi et al. (2020) also found that patients with concomitant exotropia had decreased ALFF in the parieto-occipital regions. Li et al. (2016) reported that intermittent exotropia patients showed increased neural activities in the parietal lobule during fusion stimulus. He et al. (2021) found that the intermittent exotropia patients showed decreased functional connectivity (FC) between the primary visual cortex and right cuneus and right postcentral gyrus. Yu et al. (2022) reported that strabismus patients had increased FC within the visual network and sensorimotor network. Peng et al. (2021) demonstrated that the strabismus group showed significantly decreased homotopic connectivity values in the cerebellum and frontal superior orbital. Meanwhile, the visual cortex plays an important role in the formation of stereovision. The medial temporal (MT+) plays an important role in stereoscopic depth processing. Meanwhile, the dorsal visual pathway is involved in stereoscopic depth processing. Thus, the abovementioned studies evidenced that strabismus patients were accompanied by cerebral neural activity changes in several brain regions related to vision and vision-related eye movements. However, these studies have mainly focused on static neural activities changes in strabismus patients. Recent studies reported that human brain showed dynamic neural activity. Growing neuroimaging studies demonstrated that human brain showed dynamic spontaneous neural activity, which is involved in a variety of neurophysiological functions (Liu and Duyn, 2013; Zalesky et al., 2014). However, the effect of impaired stereoscopic vision on dynamic spontaneous neural activity in patients with CE remains unknown.

The human brain shows dynamic neural activity. Dynamic neural activity is involved in higher cognitive functions, such as consciousness (Cavanna et al., 2018) and cognition (Gonzalez-Castillo et al., 2019). The ALFF method is used to assess the local intrinsic brain activity (Zang et al., 2007). The dALFF method can be used to calculate the variance of ALFF with sliding-window approaches. A sliding-window correlation analysis, where the correlation is estimated for brain activity during multiple, shows possibly overlapping temporal segments. A dALFF method is a sensitive approach for investigating dynamic brain activity (Liao et al., 2019). Previous neuroimaging studies demonstrated that the dALFF method has been successfully applied to assess the dynamic neural mechanisms of diabetic retinopathy (Huang et al., 2021), primary dysmenorrhea pain (Gui et al., 2021), and blindness (Huang et al., 2020). Thus, we hypothesized that CE may be accompanied by abnormal dynamic brain activity changes.

Based on this assumption, the purpose of the study is to investigate the dynamic neural activity changes in patients with CE. Moreover, the support vector machine (SVM) method was applied to investigate the classification efficiency using dALFF as a feature.

## Materials and Methods

### Participants

A total of 36 patients with CE and 36 healthy controls were recruited. The diagnostic criteria of patients with CE were as follows: (1) congenital CE, exodeviation angles between 15 and 80Δ. The exclusion criteria of CE individuals in the study were as follows: (1) with other ocular-related complications; (2) sensory exotropia, fixed exotropia.

### Ethical Statement

All research methods followed the Declaration of Helsinki and were approved by the Ethical Committee for Medicine of Jiangxi Provincial People's Hospital.

### MRI Acquisition

The MRI scanning was performed on a 3-tesla magnetic resonance scanner (Discovery MR 750 W system; GE Healthcare, Milwaukee, WI, USA) with the eight-channel head coil.

### FMRI Data Analysis

All preprocessing was performed using the toolbox for Data Processing & Analysis of Brain Imaging (DPABI, http://www.rfmri.org/dpabi), the more detailed steps refer to a previous study (Yan et al., 2016).

### DALFF Analysis

The dALFF method was performed using Temporal Dynamic Analysis (TDA) toolkits based on DPABI. Specifically, the R-fMRI indices mentioned above were computed with the hamming windows (window length = 30 TR, window step = 1 TR and window length = 70 TR, window step = 1). The CV (CV = SD/mean) of ALFF maps were prepared for further statistical analysis.

### SVM Analysis

The support vector machine algorithm was applied to investigate the classification. The following steps were followed: (1) the dALFF maps were selected as a classification feature. (2) Then, the SVM method was applied to classifier validation based on dALFF values in two groups. The receiver operating characteristic (ROC) curves and area under the curve (AUC) were also computed to evaluate the classification efficiency.

### Statistical Analysis

The independent sample *t*-test was used to assess the clinical scales between the two groups. The one-sample *t*-test was used to assess the spatial distribution of dALFF maps between two groups. Meanwhile, the two-sample *t*-test was applied to compare different dALFF values between the two groups (two-tailed, voxel-level *P* < 0.01, Gaussian random field correction, cluster-level *P* < 0.05).

## Results

### Demographics and Disease Characteristics

The results of these clinical data were summarized in Table 1.

**Table 1:** Clinical indicates between two groups.

**Condition**	**CE group**	**HC group**	***T*** **-values**	***P*** **-values**
Gender (male/female)	(25/11)	(25/11)	N/A	N/A
Comitant category	Congenital exotropia	N/A	N/A	N/A
Age (years)	15.80 ± 2.46	16.00 ± 2.68	−0.240	0.812
Handedness	36 R	36 R	N/A	N/A

*Independent t-test for clinical data (means ± SD). CE, comitant exotropia; HC, health control*.

### Different DALFF Values Between Two Groups

**Figure 1:**
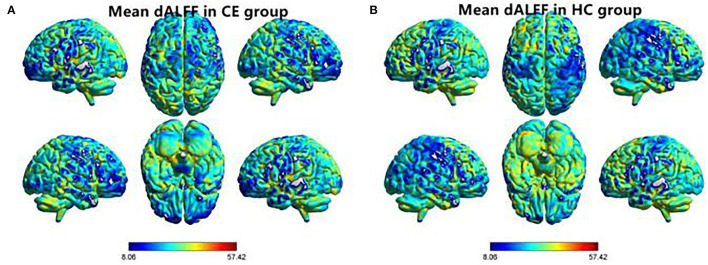
Spatial distribution of dALFF maps within CE group **(A)** and HC group **(B)**. CE, comitant exotropia; HC, health control; dALFF, dynamic amplitude of low-frequency fluctuation.

**Figure 2:**
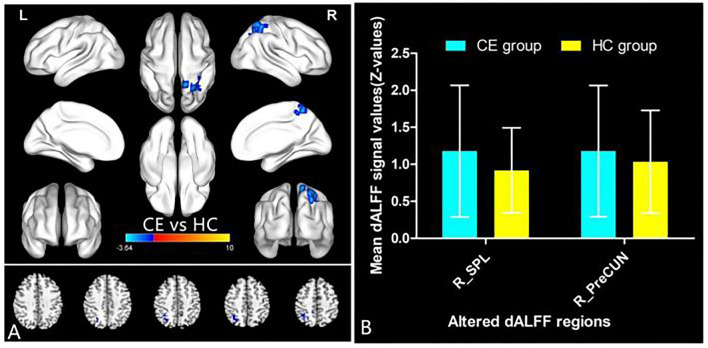
dALFF differences between two groups (window length = 30 TR, window step = 1 TR) **(A)**. The mean of altered dALFF values between two groups **(B)**. CE, comitant exotropia; HC, health control; dALFF, dynamic amplitude of low-frequency fluctuation; SPL, superior parietal lobule; PreCUN, precuneus; R, right.

**Figure 3:**
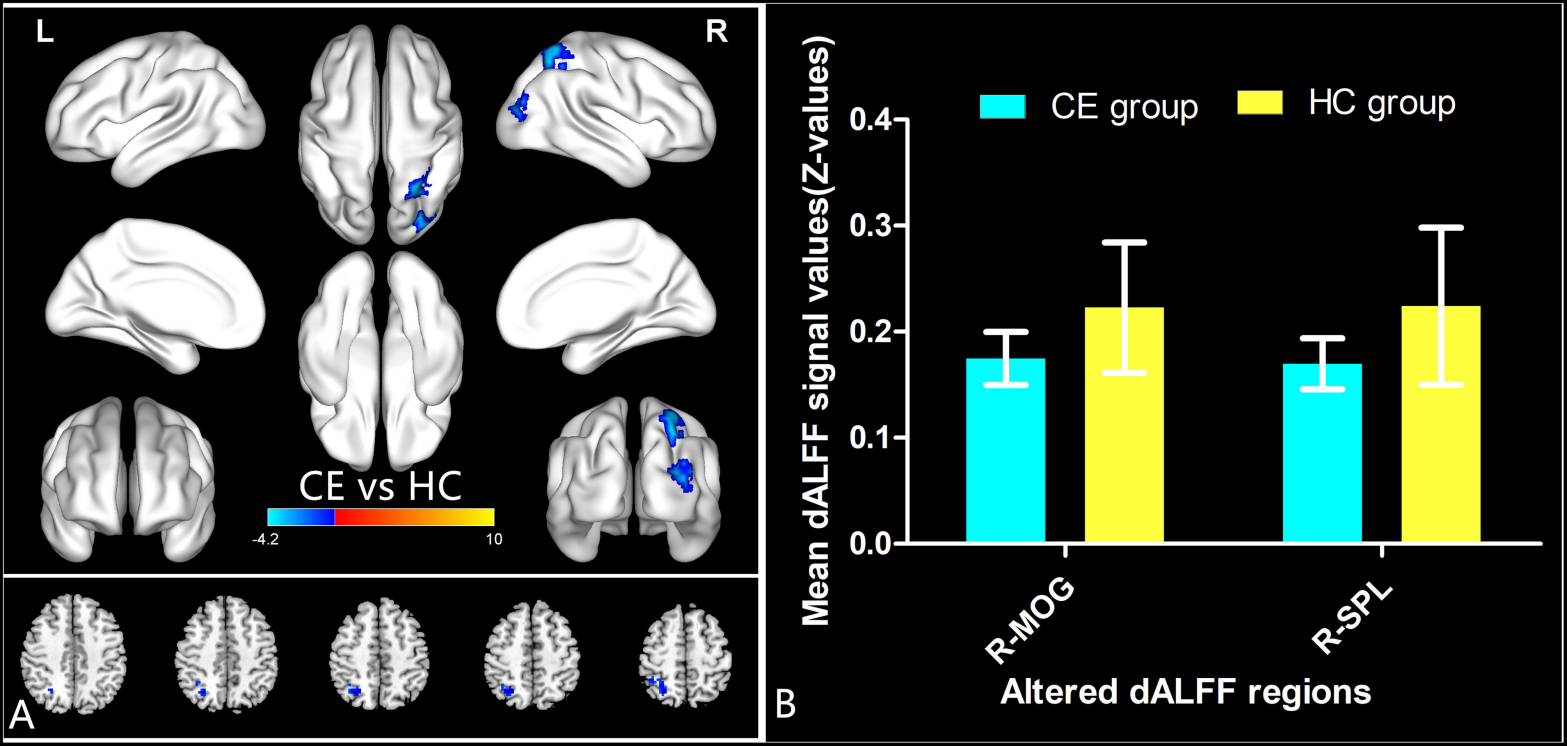
dALFF differences between two groups (window length = 70 TR, window step = 1 TR) **(A)**. The mean of altered dALFF values between two groups **(B)**. CE, comitant exotropia; HC, health control; dALFF, the dynamic amplitude of low-frequency fluctuation; MOG, middle occipital gyrus; SPL, superior parietal lobule; R, right.

The spatial distribution of dALFF maps in two groups is shown in Figures 1A,B. Compared with the HCs, patients with CE showed decreased dALFF values in the R_SPL and R_PreCUN (Table 2 and Figure 2A). The mean values of different dALFF values were shown with a histogram (Figure 2B). Compared with the HCs, patients with CE showed decreased dALFF values in the R_MOG and R_SPL (Table 3 and Figure 3A). The mean values of different dALFF values were shown with a histogram (Figure 3B).

**Table 2:** Different dALFF (window length = 30 TR, window step = 1 TR) values between two groups.

**Condition**	**Brain regions**	**BA**	**Peak T-scores**	**MNI coordinates (x,y,z)**	**Cluster size (voxels)**
CE<HC	R_SPL	–	−3.6382	39 −51 60	93
CE<HC	R_PreCUN	–	−3.4934	12 −54 66	46

*CE, comitant exotropia; HC, health control; MNI: montreal neurological Institute; dALFF, dynamic amplitude of low-frequency fluctuation*.

**Table 3:** Different dALFF (window length = 70 TR, window step = 1 TR) values between two groups.

**Condition**	**Brain regions**	**BA**	**Peak T-scores**	**MNI coordinates (x, y, z)**	**Cluster size (voxels)**
CE<HC	R-MOG	–	−4.1992	36 −78 9	76
CE<HC	R-SPL	–	−3.9941	27 −57 60	82

*CE, comitant exotropia; HC, health control; MNI: montreal neurological Institute; dALFF, dynamic amplitude of low-frequency fluctuation*.

### SVM Results

**Figure 4:**
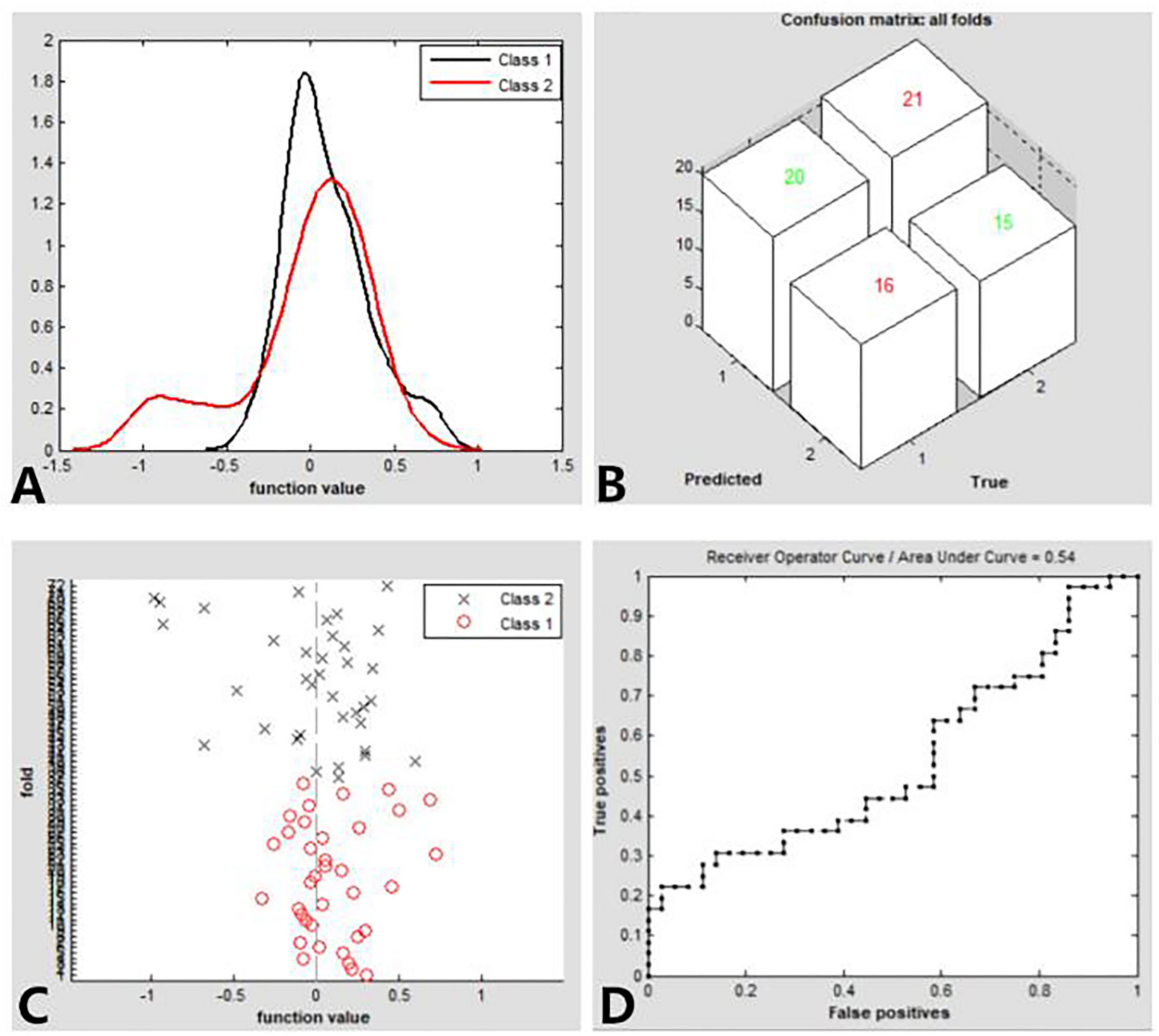
Classification results using SVM based on dALFF values. Function values of two groups (class 1: CE group; class 2: HC group) **(A)**. three-dimensional confusion matrices **(B)**. a 10-fold in the class1 and class2 **(C)**. The ROC curve of the SVM classifier with an AUC value of.54 **(D)**. CE, comitant exotropia; HC, health control; dALFF, the dynamic amplitude of low-frequency fluctuation; ROC, receiver operating characteristic; SVM, support vector machine; AUC: area under the curve.

We found that the dALFF maps showed an accuracy of 48.61% and an area under a curve of 0.54 for distinguishing the patients with CE from HCs. Figure 3 shows the classification results using SVM based on dALFF values. The function values of two groups (class 1: CE group; class 2: HC group; Figure 4A) had three-dimensional confusion matrices from machine learning analysis (Figure 4B) which have a 10-fold in class 1 and class 2 (Figure 4C). The ROC curve of the SVM classifier with an AUC value of 0.54 (Figure 4D).

## Discussion

In our study, the dALFF method was applied to investigate the dynamic neural activity changes in patients with CE. Compared with the HCs, patients with CE showed decreased dynamic neural activity changes in the right SPL and right PreCUN, which might mirror the neuropathological mechanism of stereoscopic dysfunction in patients with CE. Our study demonstrated the decreased temporal variability of dALFF in the right SPL and right PreCUN, indicating lower flexibility of these cerebral neural activities, which might reflect impaired stereoscopic vision in patients with CE.

Binocular vision can fuse visual images from the retinas of both eyes. The image is processed by the brain and formation of the stereoscopic image. The binocular disparity enables us to achieve depth perception. Thus, stereoscopic vision depends upon good vision in both eyes and good cortical mechanisms for sensory fusion. Previous neuroimaging studies demonstrated that several brain regions are involved in stereoscopic information processing related to the visual cortex (Likova and Tyler, 2007), parieto-occipital regions (Shikata et al., 1996), and the middle temporal (Uka and DeAngelis, 2004).

In our study, we found that patients with CE showed decreased dynamic neural activity changes in the right superior parietal lobule, which is involved in the discrimination of pure stereo-optic disparity information (Gulyas and Roland, 1994). Svetlana S et al. demonstrated that there were several brain regions (inferior temporal gyrus, lateral occipital sulcus) related to stereoscopic vision (Georgieva et al., 2008). Thus, our studies demonstrated that patients with CE had decreased temporal variability of dALFF in the right superior parietal lobule, which might reflect the stereoscopic dysfunction in patients with CE.

Another important finding is that patients with CE showed decreased dynamic neural activity changes in the right precuneus. The precuneus is the core component of the default mode network (DMN). The DMN is involved in emotion and cognition. Previous studies found that strabismus patients showed emotion and depression. Thus, the decreased temporal variability of dALFF in the right precuneus might reflect mood disorders in patients with CE.

There are some limitations to this study. First, we only selected 30 and 70 TR as the window length in the study. Second, our study used relatively small sample sizes. Third, the patients with CE were associated with different exotropia angles, which might be a bad influence on the result of our study.

## Conclusion

Our results showed that patients with CE had increased altered dynamic neural activity changes in the right SPL and right PreCUN, indicating lower flexibility of these cerebral neural activities, which might reflect impaired stereoscopic vision in patients with CE.

## Data Availability Statement

The data analyzed in this study is subject to the following licenses/restrictions: The raw data supporting the conclusions of this article will be made available by the authors, without undue reservation. Requests to access these datasets should be directed to Y-LZ, 804722489@qq.com.

## Ethics Statement

This study conformed with the Declaration of Helsinki and was approved by the Medical Ethics Committee of the Jiangxi Provincial People's Hospital. The patients/participants provided their written informed consent to participate in this study.

## Author Contributions

R-BC, S-YY, C-GP, and Y-LZ contributed to data collection, statistical analyses, and wrote the manuscript. All authors contributed to the article and approved the submitted version.

## Conflict of Interest

The authors declare that the research was conducted in the absence of any commercial or financial relationships that could be construed as a potential conflict of interest.

## Publisher's Note

All claims expressed in this article are solely those of the authors and do not necessarily represent those of their affiliated organizations, or those of the publisher, the editors and the reviewers. Any product that may be evaluated in this article, or claim that may be made by its manufacturer, is not guaranteed or endorsed by the publisher.
